# Emergency Medicine Residents’ Perceptions of Geriatric Emergency Medicine and Careers: A Qualitative Study

**DOI:** 10.5811/westjem.42061

**Published:** 2025-09-25

**Authors:** Katherine Selman, Abigail E. Jones, Christina Curran, Lauren Cameron-Comasco, Wendy C. Coates, Angel Li, Katren Tyler, Fernanda Bellolio, Shan W. Liu

**Affiliations:** * Cooper Medical School of Rowan University, Cooper University Health Care, Department of Emergency Medicine, Camden, New Jersey; † Harvard Medical School, Massachusetts General Hospital, Department of Emergency Medicine, Boston, Massachusetts; ‡ University of California Irvine School of Medicine, Irvine, California; § Oakland University William Beaumont School of Medicine, Corewell Health William Beaumont University Hospital, Department of Emergency Medicine, Royal Oak, Michigan; ¶ University of California Davis School of Medicine, Sacramento, California; || Geffen School of Medicine, University of California, Department of Emergency Medicine, Los Angeles, California; # The Ohio State University, Department of Emergency Medicine, Columbus, Ohio; ** Mayo Clinic, Department of Emergency Medicine, Rochester, Minnesota

## Abstract

**Introduction:**

Geriatric emergency medicine (GEM) has emerged as a subspecialty of emergency medicine (EM) with seven fellowships available throughout North America and opportunities for career development in administration, clinical leadership, education, and research. Our objective in this study was to ascertain the perspectives and understanding of the subspecialty among EM trainees.

**Methods:**

We recruited participants from four geographically diverse institutions. Three institutions were academic and had GEM faculty or divisions, and the fourth institution was a community site without geriatric-specific faculty. We conducted semi-structured interviews, adapted from a prior protocol, via teleconferencing and subsequently transcribed them. Codes were generated by two investigators and categorized into themes derived from the data.

**Results:**

Seventeen trainees with an average age of 32.1 years across four institutions participated in the study. Three themes emerged, demonstrating that trainees’ perceptions of GEM were affected by 1) education and exposure; 2) perception of geriatrics; and 3) future career considerations. Trainees with exposure to GEM had greater appreciation for the specialty, but their understanding of career opportunities was mixed. Participants acknowledged broader clinical and social considerations for older adults and in general felt that specialty training would benefit older patients. However, most participants had no personal interest in pursuing GEM, with reasons for disinterest including belief that they would only see older patients, dislike of geriatric complexity, and uncertainty about GEM as a career. Many participants identified educational opportunities for GEM, including noting that curricula include dedicated time for other subspecialties such as pediatrics but not geriatrics. Fellowship decisions were influenced by duration of training, salary, job opportunities, practice settings, and career goals.

**Conclusion:**

Emergency medicine trainees who participated in semi-structured interviews overall viewed geriatrics as an important aspect of EM with perceptions formed from exposure and education at both the institutional and individual level, perceptions of treating older adults, and future career considerations. However, interest in pursuing GEM was overall low, and participants expressed uncertainty about the subspecialty and career options, indicating opportunity for increased awareness, education, and mentorship.

## INTRODUCTION

Despite an anticipated 50% increase in demand for geriatricians due to growth of the older adult population in the United States, the number of geriatricians is projected to decrease.^1^ Older adults disproportionately use the emergency department (ED) and are more likely to receive imaging, laboratory testing, and admission to intensive care units.^2^,^3^ Furthermore, older emergency patients are more likely to have non-classic symptoms of serious diseases, polypharmacy, and cognitive impairment, considerations which if overlooked, can result in poor outcomes including inappropriate dispositions, adverse drug reactions, delayed diagnoses, or missed elder mistreatment.^4^ In response to these trends, geriatric emergency medicine (GEM) has emerged as a subspecialty for emergency clinicians to address the unique physiologic and social needs of older patients presenting for acute care.^5^

Geriatric emergency department (GED) innovations and accreditation via the American College of Emergency Physicians have expanded rapidly since publication of GED guidelines in 2013. Dedicated GEDs have been shown to decrease hospital length of stay and Medicare costs, indicating a crucial niche for emergency clinicians.^6^,^7^ In 2010, the Geriatric Competencies for EM residents were published to identify the key concepts in GEM for residents to master and to provide a basis for GEM curricula.^8^ Educational interventions based on these competencies have demonstrated improvement in geriatric knowledge and decision-making for emergency physicians at all levels of training.^9^,^10^,^11^ Seven fellowships in GEM are available in North America for further training with both one- and two-year options.^12^ Fellowship graduates in GEM have reported that fellowship training was helpful for career advancement applicable to both community and academic settings with opportunities for positions in research, education, and administration.^13^

Among emergency physicians, older patients are perceived as more difficult than younger patients with a higher burden of care.^14^,^15^ From the patient perspective, older adults’ experiences in the ED are marred by ageism and failure to accommodate for age-related changes or to address the needs of older adults.^16^ Prior research has demonstrated a perception among internal medicine trainees that geriatrics is less prestigious than other subspecialties, and there is a lack of support or enthusiasm from colleagues and mentors.^17^ While EM residents overall view older patients favorably, this attitude attenuates with each training year, and most GEM fellowship positions are unfilled each year.^18^

Despite advances in GEM and opportunities for career development within the subspecialty, little is known about EM trainees’ perceptions of the field or their experiences working with older adults, including formal GEM training during EM residency. To develop the most effective framework for training and patient care for the growing geriatric population, it is critical to understand EM trainees’ authentic experiences with and attitudes toward the aging population, the subspecialty of GEM, and factors that influence a decision to pursue a career in GEM.

**Population Health Research Capsule**

What do we already know about this issue?

*With a rising older population, geriatric emergency medicine (GEM) has emerged as a subspecialty to address the unique needs of older patients presenting for acute care*

What was the research question?

*We sought to determine EM trainees’ experiences, understanding, and attitudes toward geriatrics in GEM*.

What was the major finding of the study?

*Participants overall viewed geriatrics as an important aspect of EM, but their interest in pursuing GEM as a subspecialty was low*.

How does this improve population health?

*Improved understanding of EM trainees’ views on geriatrics provides future direction to address gaps in training and to elucidate GEM career opportunities*.

## METHODS

We followed the checklist of Standards for Reporting Qualitative Research.^19^ This was a prospective, grounded-theory, qualitative study using an interpretivist paradigm, consisting of individual semi-structured interviews of residents in postgraduate years (PGY) 2 or 3 and recent graduates at geographically diverse institutions in the United States, three with an available GEM fellowship and one community-based site. We selected upper-level residents and recent graduates as it was expected that they would be actively considering subspecialties and career paths, including graduates pursuing GEM fellowships, for insight into their career choices. Participants were identified by convenience and purposive sampling on a volunteer basis. In the recruitment letter, participants were offered a $40 gift certificate or check upon interview completion. Interviews continued until thematic saturation was reached. This project was deemed exempt by the Massachusetts General Hospital Institutional Review Board.

We adapted the interview protocol from Raj et al, 2021 (Supplement 1).^17^ The interview was divided into five sections: 1) early exposures to medicine and family support; 2) current experiences as an EM trainee; 3) career goals including subspecialty selection; 4) identity as a physician and meaning in medicine; and 5) conceptualization of GEM and ideas for generating interest in GEM. Interviews were conducted via teleconferencing by two undergraduate students AEJ and CC who had no prior relationship with participants and were trained by the study author SWL who has published multiple qualitative studies. Interviews lasted 30 to 45 minutes.

Interviews were recorded and transcribed using an automated transcription service (TranscribeMe, Inc, San Francisco, CA), and identifiable information was redacted prior to analysis. No repeat interviews were deemed necessary, and transcripts were not returned to participants. Codes were generated inductively by reading primary data and deductively from experience and grounded theory. Two study investigators KS and SWL read all transcripts independently. They then discussed common themes and ideas generated from first reading. Codes were then extracted and categorized into themes derived from the data (Figure S1).

## RESULTS

Seventeen EM trainees participated in full interviews of 114 eligible to participate. Participants had a mean age of 32.1 years, and 41.1% were female (Table 1). Fifteen participants were EM residents or recent graduates not pursuing GEM, and two participants were recent GEM fellowship graduates. Three general themes emerged from the interviews: 1) education and exposure to geriatrics; 2) perception of geriatrics and GEM as a subspecialty; and 3) career considerations.

### Education and Exposure

Patient and family exposure. Participants described various experiences that contributed to their journeys as physicians and their views on older adults. Two participants had physician parents, and three participants described their own families as influences on their medical careers and perspectives:

One of the other things growing up was relationships with my grandparents and watching them get older and then pass away eventually. That process was unique and formative. *Unidentified Interviewee* (*UI)-13*

Another participant acknowledged that they did not have this relationship with older family members and felt that this influenced their experiences with older adults as patients.

He [husband] had a wonderful relationship with his grandmother, and she was one of the most important people in his life. I think that a lot of the times when he’s interacting with older folks, there’s definitely that memory and that strong natural affinity towards older folks. I didn’t grow up with grandparents and they all passed away. So, I never really had exposure to older folks until I got to medicine. *UI-16*

Similarly, participants described the influence that patients have had on their views. In some cases, this was presented as a contrast between the EM role and the perception of what was best for the patient. This was sometimes viewed as a conflict when participants recognized that the perceived traditional EM objectives of resuscitation and interventions may not align with patient-centered outcomes.

In emergency medicine, we can have patients coming in with a code, and our goal is to resuscitate them to the best of our ability. And a lot of times, for me, personally, just getting them back is not enough. Because a lot of times, you get them back, but then they have some chronic debilitating issue that they have to deal with […]. For me, it’s very much about what does the patient want and how do we get them to that point? *UI-1*

Participants expressed the patient encounters and aspects to their jobs that help them feel fulfilled, including several emphasizing the importance of patients feeling heard and being able to connect and to communicate meaningfully with patients. Participants also described helping patients feel better, providing reassurance, finding the cause of symptoms as personally satisfying (Table 2).

Institutional education and exposure. Five of 17 participants recalled being exposed to geriatrics as a field in medical school, including both participants who completed a GEM fellowship; only one participant reported geriatrics was a mandatory rotation during medical school. Four participants described their geriatrics rotations in medical school positively. One participant tailored their residency search on availability of geriatrics programs following third-year clerkships in medical school.

In medical school, I had a fourth-year geriatrics rotation. It was medicine-based, not emergency-based, but it was a really cool rotation. We got to do a lot of home-based care. So, driving out to either assisted living facilities or patients’ homes, doing an assessment of what their home environment was like, doing medication reviews, looking at what they were on, how we could pare down medication lists. I really liked that approach to care, but I had no specific exposure to geriatric emergency care. And in fact, it wasn’t something that I knew existed until I started poking around on my own towards the end of residency. *UI-9*

Participants at the community site who did not have GEM faculty or a geriatrics division at their institution overall had less awareness of the subspecialty. These participants also report overall decreased exposure to all EM specialties.

I did rotations in the emergency department, and there were geriatric patients, but I never did an explicit geriatric emergency medicine rotation or was really exposed to that as a specialty [...]. I didn’t even know it was a specialty. *UI-4*

In general, participants who had GEM faculty or divisions described more familiarity with GEM concepts and training. One participant discussed that seeing current fellows and faculty members in subspecialties showcase their interest in turn influenced excitement and interest in the subspecialty learning. However, even among participants at institutions with GEM exposure, some trainees felt that they were still unfamiliar with GEM, particularly as a career option.

I wonder if people with a geriatric subspecialty could […] share more about what they’re doing outside of the hospital, whether that be research work or advocacy […]. I think as a resident, a lot of times there’s a little bit of mystery in terms of what a week-to-week looks like for people in various specialties. I think, in general, more light on that would be helpful. *UI-17*

Many participants at both community and academic programs identified educational opportunities for geriatrics within their training programs, noting that their curricula included defined time for other subspecialties such as pediatrics and ultrasound but not geriatrics. In some cases, this was described as a didactic opportunity, whereas another participant described an opportunity for a structured rotation like other rotations within residency:

We intentionally set aside things for pediatrics every month, but we did not specifically set aside a separate time for geriatrics and we probably should have because it’s […] more than half of our population. *UI-3*

Regardless, participants acknowledged that improved education would improve care even if trainees were not pursuing geriatrics specifically.

### Perception of Geriatrics

Broader considerations. Almost uniformly, participants acknowledged that geriatric populations have unique aspects to their care and require broader considerations in the ED. Examples of the unique aspects of the population included appropriate medication prescribing, de-escalating interventions or considering quality of life, atypical disease presentations, delirium, and disposition considerations including discharge barriers and risks of hospitalization. Ten participants (58.8%) discussed social aspects to geriatric care. For some participants, this was an aspect of care that they felt was less emphasized in their training but would be beneficial to know more about:

Beyond the medical management, we haven’t touched on other topics like disposition, like should I go to a nursing home for a SNF [skilled nursing facility] or social determinants of health as much […]. I think the more pertinent thing is their care beyond the bedside. So, we have to think about, are they safe to go home, who lives with them, who’s their healthcare proxy; if we send them home now, where did it go? Are they safe to go home? That’s a distinction. So, for older folks, you have to be more thoughtful. Not only with their medical management, but also with their post-visit needs […]. It would be good to have more emergency physicians with a more comprehensive foundation in terms of how to manage these elderly patients beyond their immediate clinical needs. *UI-6*

For one participant who had gone through GEM fellowship, addressing this complexity was a key part of improved care, but even participants not interested in GEM described the importance of a more holistic approach to the care of older adults:

[T]he role of the geriatrician is actually a lot broader than I think most people realize […]. It ends up being very all-inclusive, kind of like it would be for the pediatric population, where you have an understanding that this is a vulnerable subset of people…where we have to, as geriatricians, make sure that we are giving a whole, well-rounded and all-inclusive approach to their care. *UI-15*

Perception of geriatric EM as a subspecialty. At least one participant verbalized that they were unaware that GEM was a subspecialty available in EM. Most trainees viewed geriatric specialization in EM as necessary or beneficial for patients. Six participants specifically mentioned that having additional guidelines or algorithms for their geriatric patients would help improve their care:

I think that the specialty in general is important. The research, the literature, that can be produced that will help even the community doctor who sees the majority of these [patients] is extremely valuable. And they certainly do present different and there is a lot of nuance in the care of a geriatric patient. *UI-2*

Three participants felt that it was not necessary to have a separate subspecialty. Participants expressed that older adults constitute a large proportion of their patient population and, therefore, geriatrics is already integrated into an emergency physician’s practice without the need for specialization. One participant voiced concern that separating older adults into a separate geriatric ED could result in patients not receiving appropriately aggressive interventions. However, all three agreed that specialized geriatric care was important and beneficial to patients:

I definitely think that every emergency doctor should have training in the things that affect geriatric patients as part of the bread and butter of emergency medicine […]. I think training in geriatrics is necessary as part of emergency training and maybe constitutes a significant percentage of the content that we learn as emergency doctors. As to whether that should be separated out as its own curriculum, I’m not sure. I think certainly there are things that we need to know to take care of older adults, that we also need to know to take care of certain young adults. I would hate for it to be the case that it was so separated that only some people had that training because it is so integral to our normal practice. *UI-10*

Despite the majority agreeing that GEM is necessary and beneficial, excepting the two participants who completed GEM fellowship, no other trainees expressed interest in pursuing GEM. For some participants, they expressed no specific disinterest in geriatrics but felt more drawn to other subspecialties. Specific reasons for disinterest in GEM include a dislike for the complexity with older adults or less satisfaction after geriatric encounters. There were also perceptions of GEM as a longitudinal care model or that GEM-trained physicians would only treat older patients, and these were cited as reasons for disinterest (Table 3).

Participants had variable perceptions of the role for physicians specializing in GEM and in some cases were unsure of exact responsibilities. Trainees also described geriatric emergency physicians acting as champions and advocates for older adults:

I feel like it would be necessary, especially with more of the proportion of the population being older adults to make sure that there are people out there who are being more thoughtful and making sure that these adults are receiving appropriate care because I know there’s … ageism or elder bias. So, to help prevent or mitigate, lessen that bias, having champions for this specialized population to make sure there is a good standard of care for them as so many more people continue to age, and it becomes a larger proportion of people we take care of. UI-13

### Future Career Considerations

Fellowship. Participants cited several influences on fellowship considerations. For some participants, fellowship was not seen as necessary for employment outside an academic center. However, another participant interested in rural practice described how fellowship training in ultrasound or critical care could improve patient care in resource-limited areas. Two participants specifically referenced the ability to obtain board certification as influencing subspecialty selection. Some participants perceived that fellowship would not change their daily practice, whereas other participants expressed interest in fellowships that they felt would improve patient care:

I want a fellowship that gives me a tangible set of skills that makes me a better doctor. *UI-17*

It’s a patient population that we don’t provide the best care for in the ED, and I think there’s a lot of room with our existing science and knowledge to really improve care for these patients and make it a better experience for them. *UI-11*, on plans to pursue addiction medicine fellowship

Many participants mentioned the additional time that fellowship training requires, which would subsequently delay attending positions or require relocation:

Emergency programs are […] split between three years or four years. So, I’d already taken an extra year of residency training, which is a pay cut as opposed to doing a three-year residency and having a year of practice. Taking a fifth year to train, doing that fellowship, is an additional financial burden. I do have a wife and kid. […]. [T]hat definitely factors into the decision to extend my training is putting off another year of attending level pay. *UI-9*

Subspecialty training was also considered a way to incorporate flexibility for work-life balance in a future career. This was described in terms of having schedule flexibility, options beyond working shifts in the ED, such as working solely in a subspecialty or pursuing projects, and a proactive solution to burnout:

[O]ne day I probably may not practice emergency medicine as much and focus more like palliative care and hospice maybe in a clinic setting or a hospice setting. And so sadly, a geriatrics fellowship wouldn’t offer that same level of leaving the [ED] that I think a palliative care training would. *UI-5*

I think ultimately, for me, the calculation or the pros and cons were having a chance to carve out a subspecialty or a niche where I could have longitudinal projects or interests outside of just going in and working my shifts. Because I think my lifespan as an emergency physician, if I were to just go out into the community and work X number of shifts per month, I think I’d burn out fairly quickly. I think having dedicated training or a dedicated niche or subspecialty and the opportunity to continue projects in that area is definitely something that will help with longevity in my career. *UI-9*

Future job options and opportunities. Participants had mixed responses on future job opportunities for geriatric-trained emergency physicians. Three participants described geriatrics training as a positive influence on future job options. Others expressed uncertainty about the benefit of geriatrics specialization on job opportunities, including reservation about whether geriatrics training would impact salary.

I thought about the geriatric fellowship quite a bit, both because I think it is an important population and the nuance of the care is underappreciated. But mostly, I wasn’t sure on how employable it was, like how important it would be to employers outside of academic medicine […]. I think things that dissuaded me were how employable, like how well I could market it in employment to get jobs at particular sites that I really want over […] someone else just applying. *UI-12*

I just don’t see it as being as much of an emphasis from institutions and from hospital levels. So, I think it’s hard to […] justify for a resident with all the debt to go to do more training to not really end up in a role that you know not being able to use those skills really in a meaningful way. *UI-8*

These comments paralleled some of the discussion around the role of a geriatric emergency physician in which trainees expressed uncertainty about what opportunities would be available after specialty training and what a GEM job would entail.

## DISCUSSION

This qualitative study demonstrates that trainee interest in and perceptions of GEM are influenced by education and exposure from patients, family, institutions, and mentors; their own career goals; and their perceptions of geriatric medicine. While most participants acknowledged the importance of geriatric subspecialization within EM for patient care, only two participants were pursuing GEM as a career. Disinterest stems from competing interest with other subspecialties, complexity of older adults’ needs, and personal career goals. Leaders in GEM may consider proactively addressing more prevalent misconceptions such as that geriatric emergency physicians exclusively treat older adults.

Participants interviewed from institutions with GEM faculty had an appreciation for the subspecialty compared to participants who did not have exposure to GEM in their residency programs. This is consistent with prior work among internal medicine trainees, which demonstrated that the presence of geriatricians generated interest and an understanding of the field’s importance even among residents not interested in geriatrics.^17^ Even among three participants who felt that GEM may not be a necessary subspecialization, all agreed it was important and all trained at institutions with GEM faculty, which may have predisposed these participants to increased comfort with GEM concepts and clinical practice. Mentors in GEM may promote interest in geriatrics, support trainees, and provide an understanding of GEM as a career path. Due to limited GEM faculty nationwide, there may be opportunities to promote virtual mentorship.

There are additional opportunities for early and ongoing exposure to geriatrics through medical training. Less than one third of EM trainees in our sample had been exposed to geriatrics as a field in medical school, but experiences from those who had had a geriatrics rotation were positive and. in two cases, influenced participants’ future career paths. Geriatric rotations address the unique physiological and psychosocial challenges geriatric patients face, emphasizing patient-centered care, managing atypical presentations, polypharmacy, and discharge planning. Trainees would benefit from this education even if not pursuing fellowship, and participants in our study felt that this could be better incorporated into the curriculum in the same way that other specialties such as ultrasound are. While there are published competencies for EM residents in GEM and these have been found to align with the American Board of Emergency Medicine (ABEM) competencies, these topics are still unevenly incorporated into curricula.^8^,^20^,^21^

Access to online shared resources such as the EM clerkship curriculum’s geriatrics section, virtual GEM journal club, published simulation cases, or the GEM-focused podcast GEMCast, could provide a framework for GEM education for training institutions that do not have GEM faculty.^22^,^23^,^24^ Incorporating GEM into curricula, in the form of structured rotations, didactics specifically on geriatric domains, geriatric case review and simulation, and quality improvement projects, both enhances care quality and meets growing demand for geriatric expertise in EDs, ensuring future emergency physicians are better prepared for the increasing number of older adults they will encounter.^25^,^26^,^27^ Participants reported that formal education and board certification would legitimize GEM. Currently, GEM leaders are working with ABEM to increase geriatric content, but a focused practice designation or board certification from accrediting bodies would likely further generate interest.

Participants also lacked knowledge of GEM career opportunities. Geriatric ED accreditation is expanding across the United States, and the accreditation process requires a board-certified emergency physician to act as a champion and demonstrate geriatric education.^28^,^29^ This represents a way that institutions can prioritize and support GEM, including recruiting geriatric emergency physicians and providing protected time to spearhead geriatric initiatives. Geriatric EM is an academic discipline with viable opportunities within leadership, research, or education, as well as a focused area of clinical expertise, which trainees should be aware of when considering career development.

## LIMITATIONS

This study was conducted primarily among trainees who had exposure to GEM faculty, leading to likely oversampling; only 15% of those eligible participated in interviews, leading to likely sampling bias. We attempted to minimize social desirability bias by maintaining participant’s anonymity and employing undergraduate students to perform the interviews. No PGY-4 residents were included, which may have provided different perspectives. Only two GEM fellowship graduates participated, which limited saturation of thematic analysis in this group. As with other qualitative studies, data interpretation relies on the judgment of the investigators, and the themes identified may not capture the complexities of participants’ experiences. Qualitative studies focus on understanding specific cases rather than broad generalizations. Therefore, the findings cannot be widely generalized to all EM trainees or institutions. Future studies may be useful to assess a larger sample of EM trainees or EM-applying students with more diversity in training institutions for further insight.

## CONCLUSION

Emergency medicine trainees overall view geriatric emergency medicine as important and beneficial for patients; however, exempting GEM fellows in training, none expressed interest in pursuing GEM as a career. Geriatric exposure and education throughout medical training influence trainees’ perceptions of geriatrics, and career considerations including salary, schedule flexibility, board certification, knowledge of career opportunities, and misconceptions about GEM also impact trainees’ decisions.

## Supplementary Information





## Figures and Tables

**Table 1:** Characteristic of emergency medicine trainees who participated in a qualitative study gauging career interest in geriatric emergency medicine.

Age, mean in years	32.1
Sex, n (%)	
Male	8 (47.0)
Female	7 (41.2)
Missing	2 (11.8)
Stage in training, n (%)	
PGY-2	7 (41.2)
PGY-3	6 (35.3)
Recent graduate	2 (11.7)
GEM fellowship graduate	2 (11.7)
Residency training setting, n (%)	
Academic	12 (70.6)
Community	5 (29.4)

PGY, postgraduate year; GEM, geriatric emergency medicine.

**Table 2:** Participant perspectives on the influences of patient exposure and education.

Participants on what patient outcomes help them feel fulfilled	Addressing patient concerns and ensuring understanding	When I feel like they feel like I listen to them and address their concerns, and they understand what’s coming next. *UI-4* I think that’s why a lot of us enter emergency medicine. We want to be able to handle those life-or-death situations. And I find that empowering, and then the second is we see a lot of folks that are worried about certain things that they don’t know who to go to and…being able to provide that reassurance is meaningful to me as well. *UI-6* I think when you feel that they feel that they’ve been heard, like sometimes it’s like busy shifts and you know you’re not doing your patient justice sometimes, unfortunately like you’re just doing your best to just sort of stay afloat in a department that’s crazy busy and you’re just trying to do your best and there are times when you really feel like you’ve been able to explain everything in detail and provide all the follow-up information and resources and really walk your patient through everything and you really feel like you did right by them and that they acknowledge that and I think that’s those are probably the better moments in a sometimes challenging and really busy emergency department. *UI-8*
	Addressing the patient’s underlying problem	In an ideal world, if you could figure out what’s exactly going on, that would be the best thing. But I feel like in emergency medicine, if you can help treat someone’s pain or make sure that whatever their problem is that day is not going to kill them immediately, then I feel like I’m fulfilling my role as their emergency physician. *UI-13* What I enjoy that doesn’t always happen in EM is I really like when I have a sense of what’s happening with my patient. Sometimes the workup can be in process, but we don’t always have a clear answer of what’s going on. I find it really satisfying, though, when I can find the answer and when I know what’s causing their symptoms or their illness. I like that because then I feel more confident in the treatment that I’m giving them. And then, too, I feel more grounded in the recommendations I’m making or the discussions I’m having with family about what I think next steps may be. I find it less satisfying when somebody is really sick and we’re not sure why. I find that harder for the patient, for the family, for us. *UI-17* Definitely when I can help them on that first visit and whether or not they leave the department … feeling better. That makes me feel like I did something … Also I enjoy being able to stabilize patients and be the first one to at the very least help them in probably what is a difficult time in their life, maybe one of [the] worst days of their life. *UI-7*

Gaps in education	I think it’s a huge gap in training to not have specific geriatric principles. I don’t know that everyone needs to do a fellowship, but I think integrating more of those principles into the training pipeline, both in medical school and in residency, will only strengthen the emergency workforce. It’s not a unique thing to academic centers to have older populations. Almost every emergency department [patient population] is getting older. So, it’s definitely a skill set that will be needed if we’re going to keep up and keep providing good care. *UI-9*	

*UI*, Unidentified Interviewee.

**Table 3:** Participant perceptions of geriatric emergency medicine and reasons for disinterest.

Perception of GEM as a subspecialty	physician should have at least some […] baseline competency because this is a non-trivial portion of our patient population. We’ve seen a lot of these patients. I think you definitely need a good knowledge base to really be a good physician generally. But I also think there’s a role [for] people who have a special interest in this to help educate, make sure we’re staying up to date and, at least in my view, like designing policies, protocols, and even order sets to really care for these patients. UI-11 I think it’s built into our everyday […]. There [are] different considerations that have to be taken for that subset of the population. But in that same vein, I think that there [are] special considerations that need to be had for a lot of different types of populations, right? You could probably say the same thing about women’s health or pediatric care, right? UI-15 I think that there’s increasing need in departments around the country to have a geriatric advocate to make sure that best practices are in place in emergency departments and health systems to accommodate those much like a lot. Even emergency departments that don’t have a formal pediatric department will have a pediatric advocate or someone in the group that’s helping to ensure that best practices are being followed for that population as well. UI-12	

Reasons for disinterest in GEM as a career	Medical complexity	I would like to say that I would consider it [GEM fellowship] because I do think it’s really important. But I wouldn’t [...]. Because old people talk so much. I’m trying to get information, and it’s so hard […]. Generally, it’s a harder population for me to work with because of my personality, and the workflow of the emergency department, and … they’re complicated. They’re oftentimes, not always complicated, they’ve lived so long, so much has happened to them medically. So, when you ask them for their medical history, there’s so much to it. And just like everyone else, they don’t know what’s important. And you just get this dump of information and it’s hard. It’s like searching for a needle in a haystack sometimes to find what’s important and find it in a reasonable amount of time. UI-4
	Less satisfaction from encounters	I think one of the reasons that I like working with pediatrics so much is because, as I mentioned, I feel like they can come into the emergency department unwell, and you can see them get better during your care of them, which sometimes is only a few hours. And I just find that so satisfying. And so maybe I haven’t seen that same improvement as frequently with geriatric patients. UI-17
	Perceptions of GEM role	I don’t want to just focus on the care of older adults. I want to take care of everyone in the [ED]. I want to take good care of everyone with serious illness. So, I think that’s probably the … one driver for me to not want to do that [geriatrics]. UI-5 It would not be a good fit for me because I knew for a long time that longitudinal care was not something that I was good at. And in the world of emergency medicine, geriatric care is probably the most like longitudinal medicine that there is. UI-16

*GEM*, geriatric emergency medicine; *ED*, emergency department.
