# Supplementary material for: Emergency Medicine Residents’ Perceptions of Geriatric Emergency Medicine and Careers: A Qualitative Study
Source: West J Emerg Med. 2025 Sep 25;26(5):1404–13. doi: 10.5811/westjem.42061 (PMC12591624; doi:10.5811/westjem.42061)

Emergency medicine trainee perceptions of geriatric emergency medicine and career considerations: a qualitative study

Figure S1. Coding tree

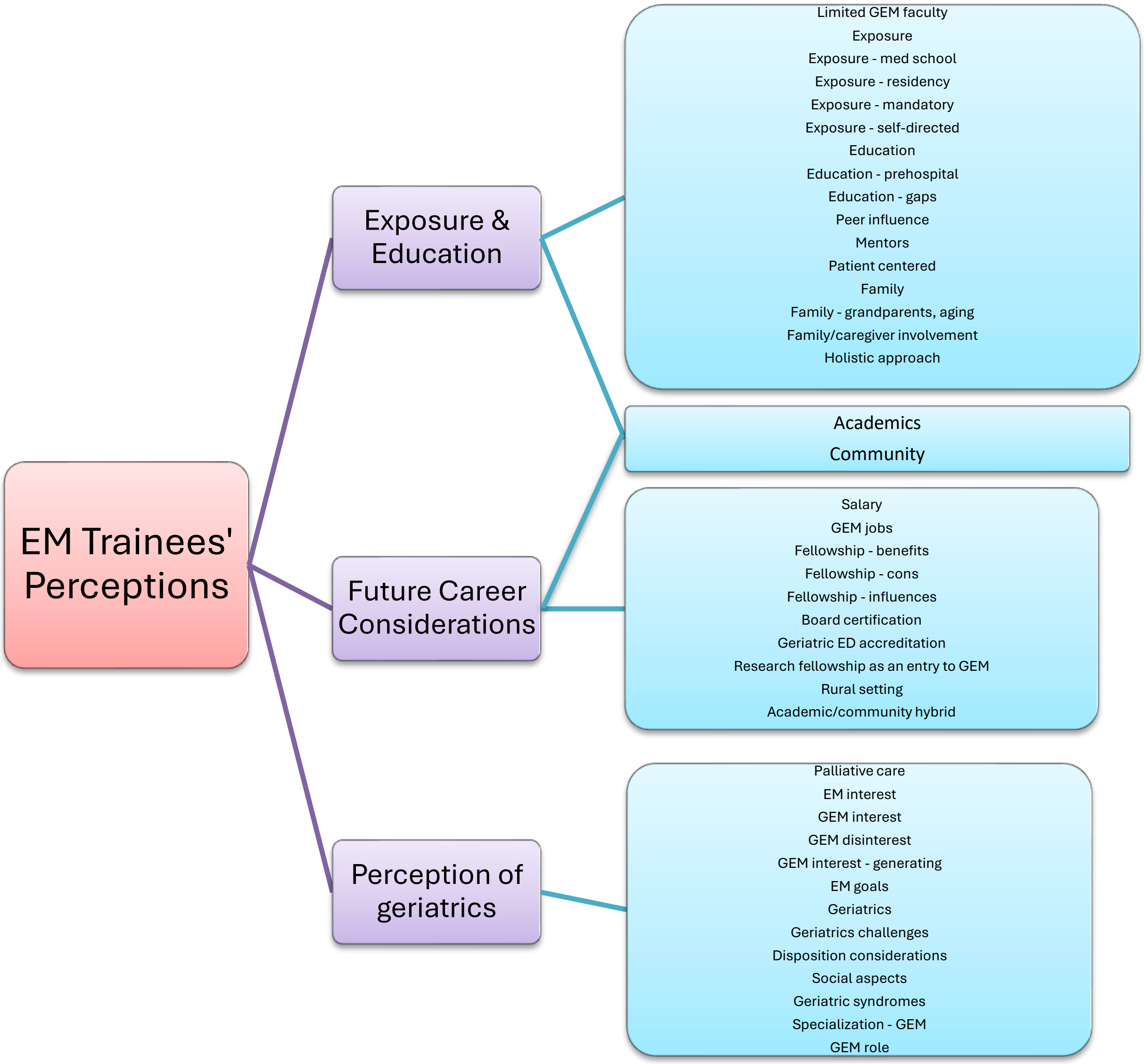

Supplement: Supplementary file 1 [file wjem-26-1404-s001.pdf]
