# Supplementary material for: Emergency Medicine Residents’ Perceptions of Geriatric Emergency Medicine and Careers: A Qualitative Study
Source: West J Emerg Med. 2025 Sep 25;26(5):1404–13. doi: 10.5811/westjem.42061 (PMC12591624; doi:10.5811/westjem.42061)
Supplement: Supplementary file 2 [file wjem-26-1404-s002.docx]

Supplemental 1. Semistructured Interview Protocol

Introduction: Thank you for taking the time to meet with me. We are studying geriatric emergency care through providers. As you may know, the population of older adults in the United States is rapidly increasing and older adult emergency department visits will similarly increase. As an emergency medicine fellow/resident/student, you offer an extremely valuable perspective as a physician who is beginning to specialize in a particular area of medicine and may choose to further subspecialize. So, I would like to ask you some questions about your experiences during your residency/school training, with particular attention to your experience with selecting a subspecialty (or how you decided not to, if that is the case), what factors are especially important in influencing your selection as you think about subspecialties, and your perspective on geriatrics emergency medicine and models of care for older adults. This interview is voluntary, and you are free to end it at any time or to skip any questions you would prefer not to answer. With your permission, I will record and transcribe our conversation, but what you share will only be seen by myself, our research team and a professional transcription service; any quotations that may be used will not include your name, program, or any information that could be identifying. The interview will take about 30-45 minutes. Shall we begin? This information is part of a research project that will be published.

Section 1. Past

- Can you tell me a little bit about your journey to becoming a physician?
- What sparked your interest in medicine?
- When did you initially become interested?
- What types of formal or informal experiences related to medicine or caregiving— e.g., volunteering, internships, family caregiving, etc.—did you engage in before joining medical school?
- Were you supported in your pursuit of a career in medicine?
- Can you tell me a little bit about why you chose emergency medicine for residency?
- Did you ever change your mind or consider another residency program (or other career) during medical school? Why?
- Was there exposure to geriatric/GEM in medical school?

Section 2. Present

- I understand from our [email/phone] conversation that you are a [Xth]-year [resident/fellow, fellowship graduate].
- Can you tell me a little bit about why you chose emergency medicine?
- How has your experience been in the residency /or fellowship program so far?
- How would you describe the level of exposure you have had to a variety of subspecialties in residency?
- How has being at [X INSTITUTION] shaped your exposure to patients across these subspecialties you mentioned above as well as exposure to mentorship?
- Who would you name as your top three mentors, and what makes them a good mentor for you?
- Are these career, personal or research mentors?
- Do you have a GEM division or department? Or GEM leader in your ED?

Section 3. Future

- What are your plans for next year? (For seniors and Fellows). (If applying to hospital or fellowship positions, “Is this an academic, community or hybrid setting?”)
- What caused you to choose this route?
- Do you plan to pursue a fellowship?
- If so, which subspecialties/fellowship are you considering and why?
- Who has been involved in your consideration of various subspecialties? - [E.g., may be family, peers]
- How supportive are your fellow residents as you consider these subspecialties? For example, maybe you have felt or feel peer pressure or that others look down on the options you are considering?

Section 4. Medical profession

- What does “being a doctor” mean to you?
- Has your perspective on this changed drastically at any point?
- What and/or who has been most influential in your perception of what it means to be a doctor?
- How do you think your definition of being a doctor is similar to or different from those of your resident/student colleagues?
- What types of tasks, activities, or practices do you look forward to engaging in as a doctor?
- What types of outcomes do you expect for your patients?
- What types of outcomes, when you observe your patients, do you believe will make you feel most that you have fulfilled your definition of being a doctor?

Section 5. Geriatrics Knowledge and exposure

- Can you describe geriatrics in just a couple sentences, perhaps including the role or responsibilities of practicing geriatricians?
- Can you describe the training you have received thus far related to geriatrics, specifically, and care of older adults, more generally?
- What are the major distinctions between and considerations for caring for older adults versus caring for adults in general (i.e., anyone > 18)? Geriatrics interest and considerations
- Do you think specialized geriatric emergency care is necessary? Why/why not?
- Do you think specialized geriatric emergency care is important? Why/why not?
- Do you feel that patients benefit from specialized geriatric emergency care? Why/why not?
- Have you ever considered subspecializing in geriatric emergency medicine? [Why or why not?]
- If yes, were/are you considering subspecializing in geriatrics to: - [Focus on GEM clinical care, GEM administration, GEM research, GEM researcher, or GEM policy]
- Can you describe what the role is for someone who completed a Geriatric EM Fellowship?
- Can you describe a few factors that have motivated your interest in geriatrics and a few factors that have discouraged you from geriatrics? - [If it does not come up naturally, ask specifically about financial incentives.] Improving programs, policies, and care
- What are some changes that [X INSTITUTION] can make to generate interest in geriatrics?
- What are some policies that will generate interest and greater consideration of geriatrics emergency medicine among physician trainees?
- What do you believe are some of the potential solutions to ensuring high quality care for older adults in the United States?
- Have you participated in the Geriatric Emergency Medicine Journal Club? (If, yes, continue with questions, otherwise, can end).
- If so, How have those meetings impacted you?
- How has your practice changed because of Geriatric EM Journal Club?

Is there anything I did not ask you about but should have?

Demographic information

- Age:
- Gender:
- Ethnicity:
- PGY year or fellow year
